# 肺癌化疗前后生活质量及抑郁情绪改变分析

**DOI:** 10.3779/j.issn.1009-3419.2011.04.10

**Published:** 2011-04-20

**Authors:** 建存 曹, 燕 王, 丽 张, 力 马

**Affiliations:** 1 300070 天津，天津医科大学研究生院 Graduate School, Tianjin Medical University, Tianjin 300070, China; 2 300052 天津，天津医科大学总医院肿瘤科 Department of Medical Oncology, Tianjin Medical University General Hospital, Tianjin 300052, China

**Keywords:** 肺肿瘤, 化疗, 生活质量, 抑郁, Lung neoplasms, Chemotherapy, Quality of life, Depression

## Abstract

**背景与目的:**

生活质量是癌症患者一个重要的观察终点，抑郁情绪更易出现在肺癌患者中。本研究旨在了解化疗对生活质量及抑郁情绪的影响。

**方法:**

随机抽取住院化疗的肺癌患者40例，分别于化疗前、化疗2周期后1周内、化疗4周期后1周内评估临床疗效，并进行EORTC QLQ-C30问卷、Zung抑郁自评量表问卷调查。

**结果:**

化疗前，生活质量功能领域、疲乏、呼吸困难条目得分较高，有抑郁情绪的占65%；化疗2周期后，化疗有效率为42.5%，认知功能条目得分上升，角色、情绪、社会功能条目得分下降，呼吸困难得分下降，疼痛、食欲不振、失眠、便秘、腹泻条目得分上升，整体生活质量下降，有抑郁情绪的占70%；化疗4周期后，化疗有效率为23%，躯体、角色、情绪、社会功能得分下降，症状领域各条目得分均上升，呼吸困难、恶心呕吐、食欲不振、经济影响条目得分上升，整体生活质量下降，有抑郁情绪的者占87.5%。

**结论:**

部分肺癌患者经化疗后症状得到缓解，但化疗过程中，抑郁情绪明显，生活质量下降，应及时评价患者生活质量及情绪改变，并给予积极心理干预，以提高患者生活质量。

## Abstract (en)

**Background and objective:**

Quality of life (QoL) is an important end point in cancer patients, and depressive symptoms are signifcantly more frequent in lung cancer patients. The aim of this study is to observe the impact of chemotherapy on quality of life and emotion among lung cancer patients.

**Methods:**

Fourty lung cancer patients were assessed with clinical outcomes, the EORTC QLQ-C30, SDS questionnaires before chemotherapy, one week afer 2 cycles of chemotherapy, one week afer 4 cycles of chemotherapy.

**Results:**

Before chemotherapy, the scores of functioning scales were high, the rate of depression was 65%. Afer 2 cycles of chemotherapy, effective rate was 42.5%, the scores of cognitive function increase, the scores of role, emotional, social function decrease, the scores of dyspnoea decrease, the scores of pain, appetite loss, insomnia, constipation, diarrhea increase, the scores of Global quality of life decrease; the rate of depression was 70%. After 4 cycles of chemotherapy, effective rate was 23%, the scores of physical, role, emotional, social function decrease, the scores of symptom scales increase, the scores of dyspnoea, nausea and vomiting, appetite loss, fnancial impact increase, the scores of global quality of life decrease, the rate of depression was 87.5%.

**Conclusion:**

Some patients have symptoms relieved, but during the chemotherapy, the patients have signifcant depression, the quality of life decrease. We should evaluate the quality of life and emotions of lung cancer patients, and give positive psychological intervention to improve the quality of life.

原发性支气管肺癌（简称肺癌）是世界上最常见的癌症，在美国支气管肺癌是肿瘤死亡的首位原因^[1]^。一般确诊时多为晚期肺癌，化疗是晚期肺癌最常用的治疗手段之一。目前临床上越来越多地关注化疗对患者生活质量的影响。本研究通过对肺癌患者化疗前后生活质量及抑郁情绪的研究，为医护人员及时发现并改善患者的情绪及生活质量提供依据。

## 1 资料与方法

### 1.1 临床资料

2009年5月-2009年10月随机选取天津医科大学总医院肿瘤科收治的已确诊的肺癌患者46例，纳入标准：①首次病理确诊的原发性支气管肺癌；②根据2009年NCCN肺癌国际分期标准为Ⅲ期-Ⅳ期；③体力评分KPS评分≥70分；④预期生存期 > 3个月；⑤愿意接受问卷调查。排除标准：①既往精神病史；②文盲者；③经解释后仍不能理解问卷条目者；④既往接受抗抑郁治疗；⑤既往酒精或药物依赖史。

### 1.2 方法

化疗前记录患者的一般资料，包括患者的性别、年龄、病理类型、疾病分期、KPS评分、化疗方案。分别于化疗前、化疗2周期后1周内、化疗4周期后1周内向患者发放欧洲癌症研究与治疗组织开发的生活质量核心量表（EORTC QLQ-C30）、Zung抑郁自评量表（Self-Rating Depression Scale, SDS），进行简单解释并告知患者回答问卷的方法。共向46例患者发放问卷，回收有效问卷（完成3次问卷者）共40例。

#### 1.2.1 EORTC QLQ-C30^[2]^

EORTC QLQ-C30是欧洲癌症研究与治疗组织推出的跨文化、跨地区面向所有癌症患者的核心量表，是目前国际通用的癌症患者生活质量测定量表。EORTC QLQ-C30包括5个功能领域、3个症状领域、1个总体健康状况领域和6个反映症状及经济状况的特异性条目，共15个领域。EORTC QLQ-C30中文版具有良好的信度、效度和反应度，可用于中国癌症患者的生活质量测定^[3]^。

#### 1.2.2 SDS量表

SDS能相当直观地反映抑郁病人的主观感受。SDS由Zung于1965年编制，1985年由我国心理学家翻译成中文，并修订取得中国常模，共20个条目，采用4级评分水平，评定主要症状出现的频率。SDS具有较高的信度及效度，其信度系数为0.92。

### 1.3 资料收集

研究者对入选患者进行跟踪调查，于化疗前、化疗2周期后1周内、化疗4周期后1周内，按WHO实体瘤疗效评价标准进行临床疗效的评价，评价标准：完全缓解（complete response, CR）、部分缓解（partial response, PR）、病灶稳定（stable disease, SD）、疾病进展（progressive disease, PD）；有效率为PR+CR人数之和所占治疗观察人数的百分率。同时患者自评EORTC QLQ-C30、SDS量表。按照各量表的计分方法计算各量表的得分。EORTC QLQ-C30量表：功能领域得分高表示功能或健康水平好，症状和整体生活质量领域得分高表示有较严重的症状和问题。SDS量表：以我国正常成人SDS标准分上限为界，SDS标准分≥50表示有抑郁症状。

### 1.4 统计分析

应用SPSS 13.0统计软件，对化疗不同阶段生活质量、抑郁情绪得分进行分析。得分如果服从正态分布，用Mean±SD表示，采用方差分析和SNK两两比较法；如不符合正态分布，用中位数表示，采用*Kruskal-Wallis*秩和检验。*P* < 0.05为有统计学差异。

## 2 结果

### 2.1 一般情况

40例患者中，男30例，女10例；年龄50岁-76岁，中位年龄61岁；腺癌14例，鳞癌11例，小细胞癌11例，低分化癌4例；Ⅲ期16例，Ⅳ期24例；化疗前KPS评分均≥70分。化疗方案：非小细胞肺癌一线化疗予GP（吉西他滨+顺铂）方案，小细胞肺癌一线化疗予EP（依托泊甙+顺铂）方案，化疗两周期后评估病情，疾病进展给予二线化疗方案（紫杉醇、培美曲塞、分子靶向药物等），其余均继续原方案化疗。

### 2.2 临床疗效

在40例患者中，化疗2周期后，CR 0例，PR 17例，有效率为42.5%；化疗4周期后，CR 0例，PR 9例，有效率为23%。

### 2.3 生活质量得分改变

肺癌患者化疗前、化疗2周期后、化疗4周期后EORTC QLQ-C30各领域得分及比较见表 1。化疗前，40例患者生活质量功能领域得分较高，疲乏、呼吸困难条目得分相对较高；化疗2周期后，功能领域认知功能条目得分上升，角色、情绪、社会功能条目得分下降，呼吸困难得分下降，疲乏症状得分下降不明显，疼痛、食欲不振、失眠、便秘、腹泻条目得分上升，经济影响得分未改变，整体生活质量下降；化疗4周期后，躯体、角色、情绪、社会功能得分下降，认知功能得分改变不明显，症状领域各条目得分均上升，呼吸困难、恶心呕吐、食欲不振、经济影响条目得分上升，失眠、便秘、腹泻条目得分改变不明显，整体生活质量下降。

**1:** 肺癌患者化疗不同时期EORTC QLQ-C30各领域得分比较（Mean±SD/Median）^★^ Compare of the scores for EORTC QLQ-C30 in different periods of chemotherapy (Mean±SD/Median)

Item	Baseline	After 2 cycles	After 4 cycles	*F*(*H*) value	*P* value
Functioning scales					
Physical	75.537±7.852	75.050±7.280	69.810±4.862^△※^	8.748	0.001
Role	64.580±23.636	61.665±18.955	52.080±18.586^△※^	4.062	0.020
Emotional	63.105±24.099	61.032±17.147	47.467±15.024^△※^	7.866	0.001
Cognitive	67.070±24.891	69.165±19.076	68.747±16.088	0.119	0.888
Social	59.995±23.216	55.415±16.194	42.067±11.337^△※^	11.197	0.001
Symptom scales					
Fatigue	48.432±23.065	47.727±19.081	52.945±16.073	0.833	0.437
Pain	22.502±14.327	32.920±19.789^☆^	34.590±19.386^※^	3.788	0.025
Nausea and vomiting	16.700	16.700	33.300^△※^	20.878	< 0.001
Specific items reflecting symptoms and economic conditions					
Dyspnoea	44.165±34.093	34.157±23.263	40.827±24.462	1.356	0.262
Insomnia	29.160±19.299	44.995±22.092^☆^	43.332±22.917^※^	4.564	0.012
Appetite loss	31.660±21.193	43.327±25.279^☆^	69.177±23.136^△※^	21.829	0.001
Constipation	0	33.300	33.300	8.313	0.016
Diarrhea	16.650	33.300	33.300	0.067	0.967
Financial impact	33.300	33.300	66.700^※^	4.283	0.017
Global quality of life					
Global quality of life	57.272±19.462	52.900±15.416	45.382±12.528^※^	5.612	0.005
^★^If the scores are normal distribution, they are expressed as Mean±SD, and use analysis of variance, SNK comparative method; if the scores are not normal distribution, they are expressed as median, and use Kruskal-Wallis rank sum test; ^☆^Compare with before chemotherapy and after 2 cycles, *P* < 0.05；^△^Compare with after 2 cycles and after 4 cycles, *P* < 0.05; ^※^Compare with before chemotherapy and after 4 cycles, *P* < 0.05.					

### 2.4 抑郁情绪改变

化疗前，40例患者有抑郁情绪的患者为26例（65%），SDS得分为51.30±6.39；化疗2周期后，有抑郁情绪的患者为28例（70%），SDS得分为52.78±5.41；化疗4周期后，有抑郁情绪的患者为35例（87.5%），SDS得分为53.87±4.73。化疗4周期后抑郁得分与化疗前相比有统计学差异（*P*=0.04），与化疗2周期后得分相比无统计学差异。

## 3 讨论

晚期肺癌是临床上最常见的恶性肿瘤之一。随着医学模式由生物医学模式向生物-心理-社会医学模式的转变，对肺癌患者的治疗目的不仅是提高生存率，更重要的是提高生命质量，关注和改善影响健康的各种躯体、心理和社会因素。本研究通过对40例肺癌患者化疗前后临床疗效的评估及生活质量、抑郁情绪的调查，发现经过化疗后，部分患者肿瘤减小，临床症状缓解，化疗使患者临床获益。但部分患者的生活质量较化疗前下降，抑郁情绪加重。以下就生活质量、抑郁情绪具体分析。

### 3.1 化疗前后生活质量比较

晚期肺癌患者生存时间较短，因此提高生存质量显得尤为重要。早在1996年，美国食品和药品管理局就把提高患者生活质量和生存获益作为新抗癌药物批准的同等重要的两个指标。本研究发现化疗前，患者生活质量功能领域得分较高，化疗2周期后，呼吸困难改善明显，但疼痛、食欲不振、失眠较前加重，生活质量有所下降。说明给予化疗后，肺癌患者局部及全身部分症状得到改善，但食欲不振加重，部分患者恶心呕吐症状加重，考虑可能的原因有：①肿瘤本身引起；②化疗药物的毒副作用也会加重患者的消化道症状；③部分患者获知病情后，会产生负性情绪及严重的心理应激，导致机体植物神经功能紊乱，造成或加重疼痛、恶心等症状。睡眠障碍考虑由癌症本身及其治疗有关的因素引起，如化疗药物本身的不良反应（胃肠道反应、乏力、脱发）及患者对化疗过程的情绪反应等。化疗4周期后，认知功能、疲乏、呼吸困难领域得分较前变化不明显，躯体、角色、情绪、社会功能下降，恶心厌食较前加重，患者感觉财务困难加重，总体生活质量呈下降趋势。肺癌患者的身体状况和反复住院治疗均妨碍患者的家庭生活和社交活动，社会价值受到影响。另外，昂贵的医疗费用与患者有限的经济支付能力成为治疗过程中较为突出的一对矛盾，经济状况也是导致肺癌患者化疗生活质量低的重要因素。本研究中患者疲乏领域得分变化不明显，但Dagnelie等^[4]^对64例接受高剂量放疗的肺癌及乳腺癌患者进行生活质量调查，发现在生活质量所有领域里，疲劳、乏力是影响整体生活质量的主要因素。

### 3.2 化疗前后抑郁情绪比较

心理因素在癌症发生、发展及预后中的作用越来越受到重视。伴有抑郁、焦虑的人群发生肺癌的风险高于其它肿瘤^[5]^，而且有研究^[6]^发现癌症患者中抑郁情绪的发生率明显高于正常人，严重影响化疗患者的生活质量^[7]^，须引起临床医护人员的高度重视。

抑郁是一种以心境低落为主要特点的情绪反应，肺癌患者初次确诊时抑郁的发病率就很高^[8]^。本研究发现随着化疗周期的增加，肺癌患者抑郁情绪逐步加重。研究^[9]^分析癌症患者产生一系列心理变化的原因有4个方面：①患者对疾病和住院的应激反应；②合并精神疾患，如焦虑或抑郁症；③疾病导致继发性情绪障碍；④某些药物的副作用，如抗癌药物不良反应等。抑郁情绪可引起体内自主神经调节紊乱，引发一系列的生理病理改变，如儿茶酚胺的过量分泌、脂类代谢紊乱、各种促凝物质和有强烈血管收缩作用的血栓素A2的释放、心率加快和血压上升等，其结果是加重肺癌患者的躯体症状，降低生活质量以及导致看病次数增加、住院时间延长、治疗依从性差及增加医疗费用等^[9]^。抑郁状态是肺癌发病前的重要危险因素之一，同时也是肺癌发病后的主要负性情绪表现^[10-12]^，它不仅影响着患者的近期临床疗效，也决定着患者的预后和转归^[13-15]^。

总之，化疗能够改善肺癌患者部分临床症状，使部分肺癌患者临床获益，但随着化疗周期的增加，躯体、社会等功能下降，抑郁情绪明显，总体生活质量降低。大多数肺癌诊断时即为晚期肺癌，不能手术切除，因此生活质量是我们选择治疗方案时需要考虑的一个主要因素^[16]^。本研究由于观察时间有限，且观察的病例数较少，故对观察患者的长期心理变化过程还不能做出评价，因此，更确切的结果有待于更进一步深入、细致的研究。
